# The art of persuasion - is it ethical to influence patients?

**DOI:** 10.1192/j.eurpsy.2023.189

**Published:** 2023-07-19

**Authors:** F. Prates

**Affiliations:** Psychiatry, Centro Hospitalar de Lisboa Ocidental, Lisbon, Portugal

## Abstract

**Abstract:**

In this third session of our motivational interviewing workshop, we address the art of persuasion and what to do and not do in the clinical setting. The change process in a motivational interviewing setting is based on collaborative principles between the patient and the therapist and is not imposed. We show examples of what to do and not to do with the previously learned techniques.

**Disclosure of Interest:**

None Declared

